# Beyond S-cross section design: mechanical properties, metallurgical features and shaping ability of two nickel-titanium rotary instruments in double curved canals

**DOI:** 10.1007/s00784-026-06953-1

**Published:** 2026-05-29

**Authors:** Larissa Tres, Gabriel Barcelos Só, Raimundo Sales de Oliveira Neto, Marco Antônio Húngaro Duarte, Carolina Horn Troian-Michel, Ricardo Abreu da Rosa, Murilo Priori Alcalde, Marcus Vinícius Reis Só

**Affiliations:** 1 Department of Conservative Dentistry, School of Dentistry, Rio Grande do Sul Federal University (UFRGS), R. Ramiro Barcelos, 2492 - Santa Cecília, Porto Alegre, RS 90035-004 Brazil; 2 Department of Stomatology, Faculty of Dentistry, Federal University of Santa Maria (UFSM), Santa Maria, RS Brazil; 3 Department of Operative Dentistry, Endodontics and Dental Materials, Bauru School of Dentistry, University of São Paulo, São Paulo, Brazil

**Keywords:** Differential scanning calorimetry, Endodontics, Microcomputed tomography, Cyclic fatigue, Scanning electron microscopy

## Abstract

**Objectives:**

This study evaluated the metallurgical properties and shaping ability of two nickel-titanium instruments in artificial double-curvature canals.

**Methods:**

20 NiTi instruments (25 mm in length) with *S*-shaped cross-section design and different NiTi alloy were selected for analysis (*n* = 10, SPIN 25.06; *n* = 10 Rotate 25.06). The instruments were evaluated with respect to geometric design, differential scanning calorimetry, and shaping ability. Cyclic fatigue was also evaluated after three simulated clinical uses, comparing it to that of new instruments. Data were analyzed using parametric and nonparametric tests according to data distribution, with a significance level set at 5%.

**Results:**

The instruments exhibited similar design features and different phase transformation temperatures. Rotate 25.06 presented lower metal mass volume and cross-sectional area than Spin 25.06 (*P* < 0.05). Rotate 25.06 resulted in lower canal deviation and better center ability than Spin 25.06 (*P* < 0.05). Spin 25.06 instruments demonstrated greater cyclic fatigue resistance (*P* < 0.05); however, after three simulated clinical uses, both instruments exhibited similar clinical fatigue resistance (*P* > 0.05).

**Conclusion:**

Although Spin 25.06 showed greater resistance to cyclic fatigue and better phase transformation temperature, Rotate 25.06 achieved superior shaping performance, promoting less deviation and better centering ability in the artificial double-curved root canals.

**Clinical relevance:**

Selection of NiTi instruments must consider cyclic fatigue resistance, shaping ability and metallurgical behavior. Our in vitro findings highlight the need of balancing instruments’ properties to reduce procedural errors and optimize clinical outcomes.

## Introduction

Root canal preparation is an important step in endodontic therapy, as widening the canal allows the flow of irrigating agents [1, 2] and enables adequate disinfection of the root canal. In the presence of curvatures, root canal preparation becomes more challenging due to the tendency of different preparation techniques to deviate from the original root canal bed [3]. This deviation, when in the apical portion, can result in foraminal displacement, step formation or perforations [4] .

Nickel-titanium (NiTi) instruments have been manufactured using alloys with different heat treatments [5, 6], which allows clinicians to overcome the shortcomings of stainless-steel instruments [7] and’ reduce procedural errors during instrumentation [8]. However, even with fewer procedural errors, the risk of fracture of a NiTi instrument inside the root canal is still a concern for clinicians, especially in teeth with varying degrees of curvature. Regardless of the kinematics employed, these instruments can suffer from torsional stress and cyclic fatigue. Fracture due to torsion might happen when a part of the instrument binds in the root canal while the handpiece continues to rotate at a constant speed. When instrument’s elastic limit is exceeded, its fracture occurs [9]. The torsional resistance of rotary instruments is highly affected by their polar moment of inertia, which is a measure of an object’s ability to resist torsional stress when an amount of torque is applied to it [10]. On the other hand, cyclic fatigue due to bending occurs due to repeated compressive and tensile stresses when the instrument rotates in a curved canal [11, 12].

To understand an instrument’s performance and select it for challenging root canal curvatures, careful analysis of various methods is essential. These methods evaluate modeling capacity, including apical deviation, centering capacity and post-preparation volume. They also assess mechanical properties, such as cyclic and torsional fatigue. Finally, it’s crucial to study the metal core volume and section design of these instruments for use in medium and high complexity curvatures [12, 13].

Recently, the Spin (MK-Life) was introduced to the market based in VDW Rotate instruments, both with rotary kinematics. The VDW Rotate instruments present constant taper, manufactured using a heat-treated NiTi alloy and an S-shaped cross-Sect [12]. NiTi Spin instruments feature their own heat treatment (Control Memory), constant taper and electrolytic plasma polishing [14] as well as an S-shaped cross-section. According to Kim et al. [15], endodontic files with this shape have a smaller metal core and, consequently, greater fatigue resistance. The aim of this study is to evaluate the metallurgical features, shaping ability and mechanical properties of two instruments with the same taper (25.06), same cross-section, and different heat treatments.

## Material and methods

### Study design

This is an in vitro study, with blinding for the evaluators. The factors under study were apical deviation, centering ability, prepared canal volume, cyclic fatigue of new instruments and after 3 uses, metal mass volume and cross-sectional analysis.

### Design assessment

#### Metal mass volume and cross-sectional area analysis

Twenty rotary NiTi instruments (*n* = 10 SPIN file #25.06; MK Life, Porto Alegre, Brazil, and *n* = 10 ROTATE file #25.06, VDW GmbH, Munich, Germany) were analyzed. Metal mass volume (mm^3^) and cross-sectional area (µm^2^) were assessed using micro-computed tomography (micro-CT) (Skyscan 1174v2; Bruker Belgium N.V., Kontich, Belgium), following a previously described methodology [16, 17]. Micro-CT imaging was performed using the following parameters: 50 kV, 800 µA, 360° rotation, isotropic resolution of 14.1 μm, and a 0.5 mm-thick aluminum filter. The acquired images were reconstructed into cross-sectional slices perpendicular to the instrument’s long axis using dedicated software (NRecon ver. 1.6.3, Bruker Belgium N.V.), allowing both two-dimensional (2D) and three-dimensional (3D) analyses. In the 3D analysis, metal mass volume was measured from the instrument tip to the 6th and 10th mm, which correspond to the locations of the two curvatures. For 2D analysis, reconstructed cross-section slices provided a topographic view at 6 and 10 mm from the tip, where the cross-section area (µm^2^) was quantified for comparison.

#### Evaluation of active blades

Seven instruments from each group were randomly selected for examination under a stereomicroscope (Opmi Pico, Carl Zeiss Surgical, Jena, Germany) using ImageJ software (ver. 1.50e; Laboratory of Optical and Computational Instrumentation, Madison, WI, USA) at 13.6× magnification [16]. The images were analyzed to assess the length of the active cutting blade, number of spirals, spirals per millimeter, and spiral direction. For illustrative purposes, the instruments were also photographed using a digital camera (Canon EOS 500D; Canon, Tokyo, Japan) paired with a 1:1 Macro Lens (IRIX 150 mm F/2.8; TH Swiss, Baar, Switzerland) to ensure high-resolution images with minimal distortion, as performed by a previous study [16] .

### Differential scanning calorimetry (DSC)

Differential scanning calorimetry (DSC) tests were performed to assess the phase transformation temperatures of the tested instruments (*n* = 3 per group), as performed by two previous studies [12, 16]. This standardized protocol ensures reliable measurement of phase transitions by monitoring heat flow variations as a function of temperature. Small fragments measuring 3 to 5 mm in length and weighing 7 to 12 mg were extracted from the active blade of each instrument and etched in a solution composed of 25% hydrofluoric acid, 45% nitric acid, and 30% distilled water for 2 min. After rinsing with distilled water, the specimens were placed in aluminum pans, with an empty pan serving as a control. The heat cycle lasted 45 min and included: an isothermal hold at 25 °C for 5 min, heating to 150 °C (10 °C/min), a 2-minute isothermal hold, cooling to -30 °C (10 °C/min), another 2-minute isothermal hold, reheating to 150 °C (10 °C/min), and a final isothermal hold for 2 min, followed by cooling to 25 °C. DSC analyses were conducted using the DSC Stare 1 system (DSC 204 F1 Phoenix; Netzsch-Gerätebau GmbH, Selb, Germany). The resulting phase transformation temperatures were processed with Netzsch Proteus Thermal Analysis software (Netzsch-Gerätebau GmbH, Selb, Germany). To ensure reproducibility, each test was performed twice per group, and results were compared for consistency.

### Micro-CT assessment of preparation

The sample size was calculated using Biostat 5.0 software. An alpha error of 0.05, a beta power of 0.80, and an N2/N1 ratio of 1 were stipulated. A total of eight samples per group was indicated as the ideal size necessary to find significant differences. However, 10% of the total number of samples were added to compensate for possible outliers that could lead to sample loss, establishing a sample size of 9 teeth per group.

A total of 18 artificial canines made of resin (IM, Curitiba, Brazil) with double curvature and 12 mm in length, were used. The curvatures were located at the 6th and 10th mm from the apex. The samples were scanned using a computerized microtomograph (SkyScan1174v2; Bruker-micro-CT, Ethiolles, Belgium) at maximum power according to the following parameters: 50 kV; 800 µA, with a voxel size of 28.24 µA. Images with 512 × 652 pixels were obtained with acquisition intervals of 0.7°, totaling 360°. The image sequences were reconstructed using the SkyScan 6.4.8 program.

#### Canal preparation

The canals of all teeth were explored with C-pilot files (VDW) size #10, up to the foraminal limit. The working length (WL) was 1 mm short of the apical foramen and was established by manually visualizing the tip in the foramen. The teeth were then subjected to mechanical root canal preparation. The roots were randomly distributed (http://www.random.org/integers/) into two groups (*n* = 9) according to the rotary system that was used.

Initially, a glide path was performed with the #15 manual file (C pilot, VDW). Next, cervical and apical preparation were performed using the following rotary instruments:

- SPIN GROUP − 9 teeth - SPIN file (MK Life, Porto Alegre, Brazil). The canal was irrigated with 5 ml of 2.5% NaOCl, and canal preparation was performed using the sequence recommended by the manufacturer. The #17.12 instrument (MK Life, Porto Alegre, Brazil) was responsible for preparing the cervical third and was coupled to an electric motor (VDW GmbH, Munich, Germany) and used in a rotary motion at 350 rpm and 2 N of torque. For this instrument, three entry and exit movements were performed only in the cervical third (4 mm). The instrumentation was followed using instruments #15.04, 20.05, and #25.06 in a rotary motion with three pecking movements in the apical direction, removal, cleaning of the instrument, and successive repetitions until reaching the CT. The instrument was coupled to an electric motor (VDW GmbH, Munich, Germany) and used at 350 rpm and 2 N of torque.
- VDW ROTATE GROUP − 9 teeth - ROTATE file (VDW GmbH, Munich, Germany). The procedure was performed with the same sequence and protocol used in group 1.

During root canal preparation, the canals were irrigated with 5 ml of 2.5% NaOCl using the syringe-needle irrigation technique after each instrument removal [18, 19]. The purpose of this irrigation was to remove resin debris and lubricate the root canal during its preparation. After root canal shaping, the instruments were ultrasonically cleaned for 5 min and sterilized at 121 °C, at a pressure of 30 psi, for 20 min. Each instrument was used on 3 teeth.

After completion of the biomechanical preparation of the root canal, the canals were irrigated with 5 ml of saline solution, dried with #25 absorbent paper tips, and subjected to microcomputed tomography (micro-CT 2) using the same parameters as initially used.

#### Evaluation of apical deviation and centering ratio

Apical deviation and centering capacity were evaluated using Analyzer software (Skyscan; Kontich, Belgium) at three root levels, 2, 3, and 4 mm from the apex. A calibrated evaluator measured all values. Both results were determined by the shortest distance from the canal margin to the outer part of the root (mesial and distal) before and after canal preparation.

The following formulas for apical deviation and centralization ratio were used [(m1-m2) – (d1-d2)] and (m1-m2)/(d1-d2) or (d1-d2)/(m1-m2), respectively. Where m1 and m2 were the shortest distances from the mesial root margin to the mesial margin of the canal before and after preparation, respectively; and d1 and d2 were the shortest distances from the distal root margin to the distal margin of the canal before and after preparation, respectively. A result other than 0 meant canal transport, and a result equal to 1 represented perfect centering [20] .

#### Canal volume assessment

The pre- and post-instrumentation images were superimposed using the 3D recording function of DataViewer v.1.5.1 software (Bruker micro-CT). The recorded images were processed in CTAN v.1.14.4 software (Bruker micro-CT) to calculate the apical volume, comprising the last four apical millimeters, and the total volume, which consisted of the canal volume extending from the root apex to 12 mm in the cervical direction.

### Cyclic fatigue test

Static cyclic fatigue tests were performed using a custom device that allowed reproducible simulation of an instrument confined in a curved artificial canal at room temperature [17, 21]. The artificial canal was manufactured to reproduce the size and taper of the instrument, thus providing the instrument with an adequate trajectory with a curvature angle of 60° and a curvature radius of 5 mm [22]. The curvature of the stainless-steel artificial canal was adapted to a guide cylinder made of the same material (curvature angle of 60°; radius of 5 mm). The arch had a 1 mm deep groove located 5 mm from the top to match the height of the contra-angle. The groove served as a guide path for the instrument, which remained curved and free to rotate between the cylinder and the outer arch.

Ten instruments of each brand (5 new and 5 after 3 uses on acrylic teeth with double-curved canals) were activated using a 6:1 reduction handpiece powered by a torque-controlled motor (Elements Motor) using the predefined programs “CUSTOM MODE” to activate Spin #25. 06 and VDWRotate #25.06. The “CUSTOME MODE” was used at 300 RPM and 2 N of torque. To reduce friction of the instrument when it met the artificial canal walls, a special high-flow synthetic oil prepared for lubrication of mechanical parts (Super Oil, Singer Co Ltd, Elizabethport, NJ) was applied. The number of cycles until instrument fractures was recorded and stopped as soon as a fracture was detected. During this stage, a video recording was made simultaneously, and the recordings were observed to ensure the precise time of instrument fracture.

#### Scanning electron microscopy (SEM)

The fractured fragments of cyclic test of each experimental group were examined using a Scanning Electron Microscope (SEM – JSM-TLLOA; JEOL, Tokyo, Japan) to determine the topographic feature of fractured fragments at 150X magnification. The cross-sectional configuration of instruments and the core diameter were evaluated using a dedicated software (AutoCAD, Autodesk InC, San Rafael, CA), following a methodology similar proposed by Burklein et al. [23].

### Statistical analysis

The evaluation of instrument design parameters, such as the number of blades and helical angles, was purely descriptive and therefore not subjected to statistical analysis. The normality of the data from the metal mass volume and cross-sectional area measurements was confirmed using the Shapiro-Wilk test. Group comparisons were then performed using an independent Student *t*-test, with a significance level set at 5% (IBM SPSS ver. 22.0 for Windows; IBM Corp, Armonk, NY, USA).

Due to the non-normal distribution of apical deviation values, the Mann Whitney test was used in the analysis of apical deviation between groups, while the Wilcoxon test was used in the intra-group analysis. In the analysis of centralization, volume, and cyclic fatigue, the data were analyzed using the unpaired t-test.

## Results

### Design assessment

**Fig. 1:**
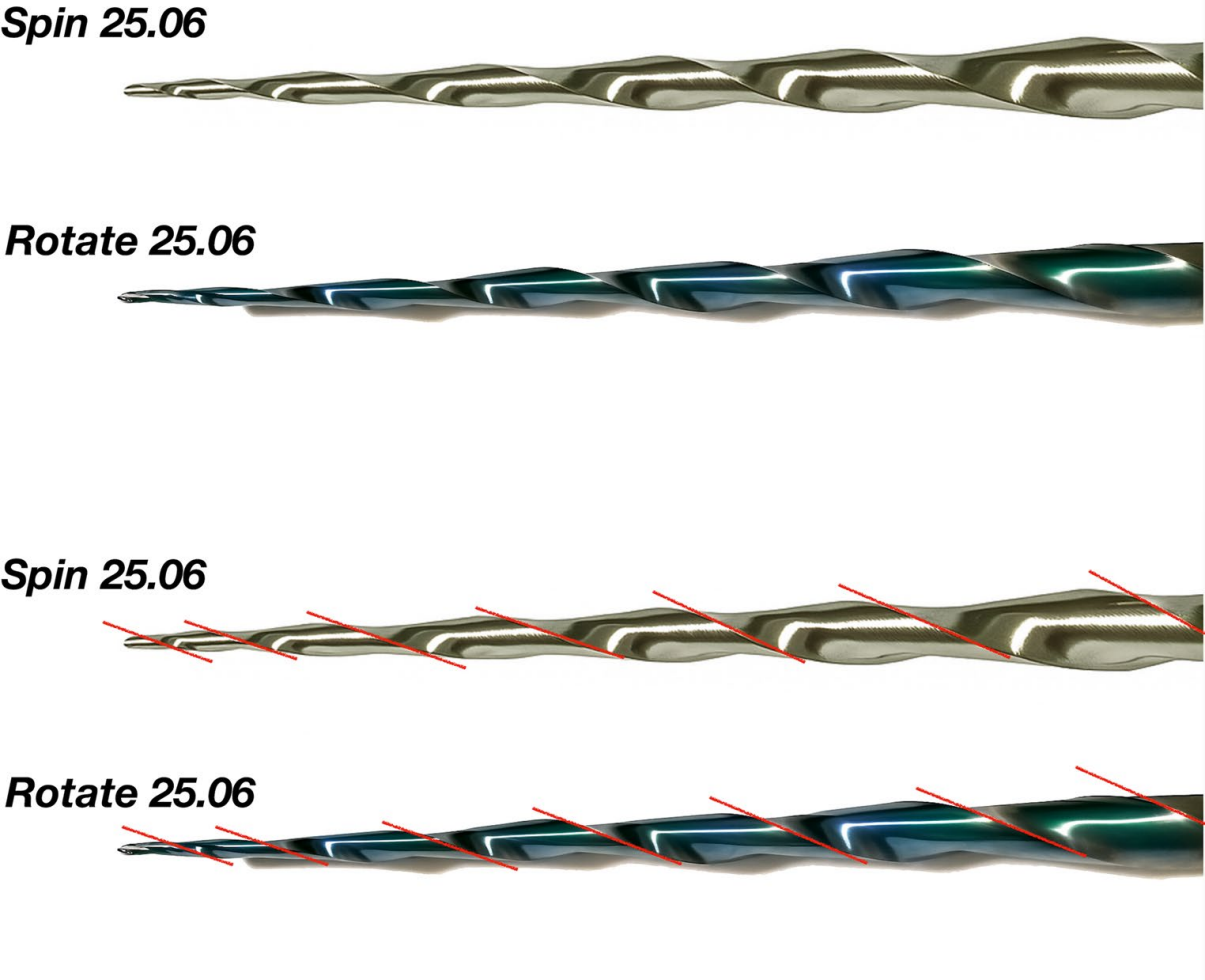
Geometric design characteristics of the tested instruments

The geometric design characteristics of the tested instruments are illustrated in Fig. 1. The active blade length remained consistent at 16 mm for both instruments. The number of spirals was 7 along the 16 mm, which provided 0,43 spirals per millimeter for both. Additionally, the helical angle presented a smooth difference, with the Spin 25.06 displaying the mean helical angle 21.9°, while for the Rotate 25.06 the mean was 22.1°.

The results of metal mass volume (mm^3^) and cross-sectional area (µm^2^) analyses are presented in Table 1; Fig. 4(B). The Rotate 25.06 instruments exhibited a significantly lower metal mass volume and cross-sectional area at both 6 mm and 10 mm from the instrument’s tip compared to Spin 25.06 (*P* < 0.05).

**Table 1:** Mean values and standard deviations of metal mass volume (mm^3^) and cross-section area (µm^2^) at 6 and 10 mm from the tips of the instruments tested

	Metal mass volume		Cross-section area	
	6 mm	10 mm	6 mm	10 mm
Rotate 25.06	0.31^a^±0.08	0.62^a^±0.07	84^a^±0.81	167.4^a^ ± 0.84
Spin 25.06	0.43^b^±0.08	0.81^b^±0.09	95.1^b^ ± 0.83	181.3^b^ ± 0.82

Different superscript letters in the same column indicate statistical differences between groups (P<0.05)

### Differential scanning calorimetry analysis (DSC)

**Fig. 2:**
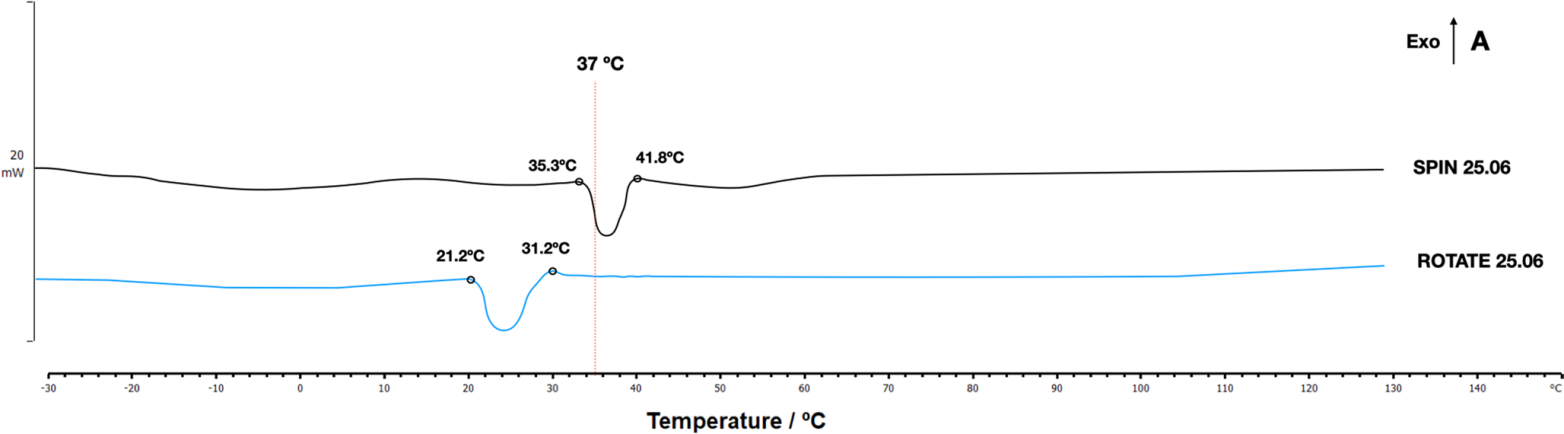
DSC charts illustrating the phase transformation temperatures. The austenite starts (As), and austenite finish (Af) temperatures are presented in the heating curve (left to right)

The DSC curves revealed distinct phase transformation temperatures between the Spin 25.06 and Rotate 25.06. The austenite start (As) and austenite finish (Af) temperatures are presented in the heating curve (left to right). The Rotate 25.06 presented As and Af temperature of 21.2 °C and 31.2 °C, respectively, whereas the Spin 25.06 showed As and Af of 35.3 °C and 41.8 °C, respectively (Fig. 2). Considering the temperatures commonly used at the cyclic fatigue are room temperature (24 °C) and the body temperature (36 °C), Rotate 25.06 instruments would display a mixed microsctructure of Austenite and R-phase at room temperature and a fully Austenitic microstructure at body temperature. In contrast, the Spin 25.06 instruments would predominantly present the R-phase at room temperature and mixed microstructure of Austenite and R-Phase at body temperature.

### Canal transportation and centering ability

There were no significant differences between the rotary systems in terms of canal deviation to the right side at the 2-, 3-, and 4-mm levels (*P* > 0.05). When the left side was considered, the Spin system promoted greater deviations at the 2-, 3-, and 4-mm levels (*P* < 0.05). In an intra-group analysis for both rotary systems, the greatest deviation always occurred on the left side, regardless of the levels evaluated (*P* < 0.05).

**Fig. 3:**
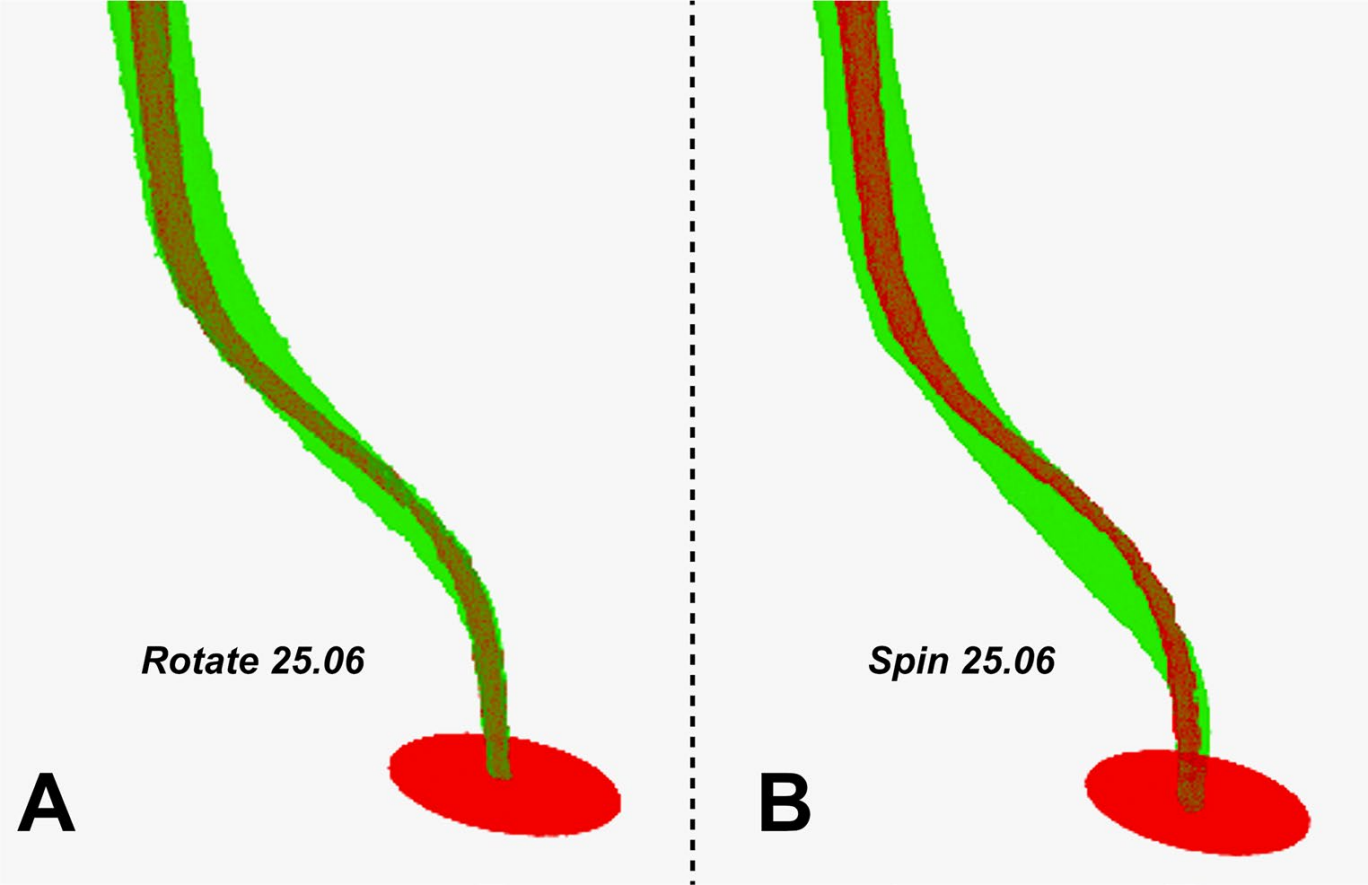
3D reconstructions of experimental groups. A- Rotate 25.06; B-Spin 25.06. In red, the original anatomy of the root canal (pre-operation) and in green the final instrumentation with size #25.06 instrument (post-operation)

The Rotate system showed better centering ability than the Spin system (*P* = 0.0003). Regarding volume, when the two systems were compared, the Spin system promoted higher volume values (*P* < 0.0001) (Table 2; Fig. 3).

**Table 2:** Apical deviation values (at 2, 3, and 4 mm), centering ability, pre- and post-preparation volume (mm), and number of cycles until fatigue of the instrument with 0 use and after 3 uses

		Apical deviation (mm)	Centering ability	Volume (mm^3^)	Cyclic fatigue new	Cyclic fatigue 3 uses
		Median	Mean (SD)	Mean (Pre/Post)	Mean (SD)	Mean (SD)
SPIN			2,928^A^ (0,532)	8,310 ^a^ /19,72^b^	1669^aA^ (118)	1222^bA^ (332)
	2 mm	R- 0,07* L -0,22**				
	3 mm	R- 0,04* L- 0,23**				
	4 mm	R- 0,07* L- 0,30**				
ROTATE			0,522^B^ (0,198)	8,310^a^ / 17,57^b^	1068^aB^ (203)	1230^aA^ (229)
	2 mm	R- 0,15* L- 0,06**				
	3 mm	R- 0,10* L- 0,06**				
	4 mm	R-0,15* L-0,12**				

*Represents no statistical differences between systems when comparing apical deviation to the right side between equal mm

**Represents statistical differences between systems when comparing apical deviation to the left side between equal mm. Small lowercase letters in the row indicate statistical differences in volume and fatigue parameters

Different capital letters in the column indicate statistical significance between systems in the parameters of centralization capacity and cyclic fatigue (P<0.05)

### Cyclic fatigue

The cyclic fatigue resistance test of new instruments demonstrated that the Rotate system showed a significantly lower number of cycles to fracture than the Spin system (*P* < 0.05). Regarding resistance to fatigue after 3 uses, the number of cycles to fracture was similar for both systems (*P* > 0.05). The Spin system (without use) showed greater resistance to cyclic fatigue than after 3 uses (Table 2).

**Fig. 4:**
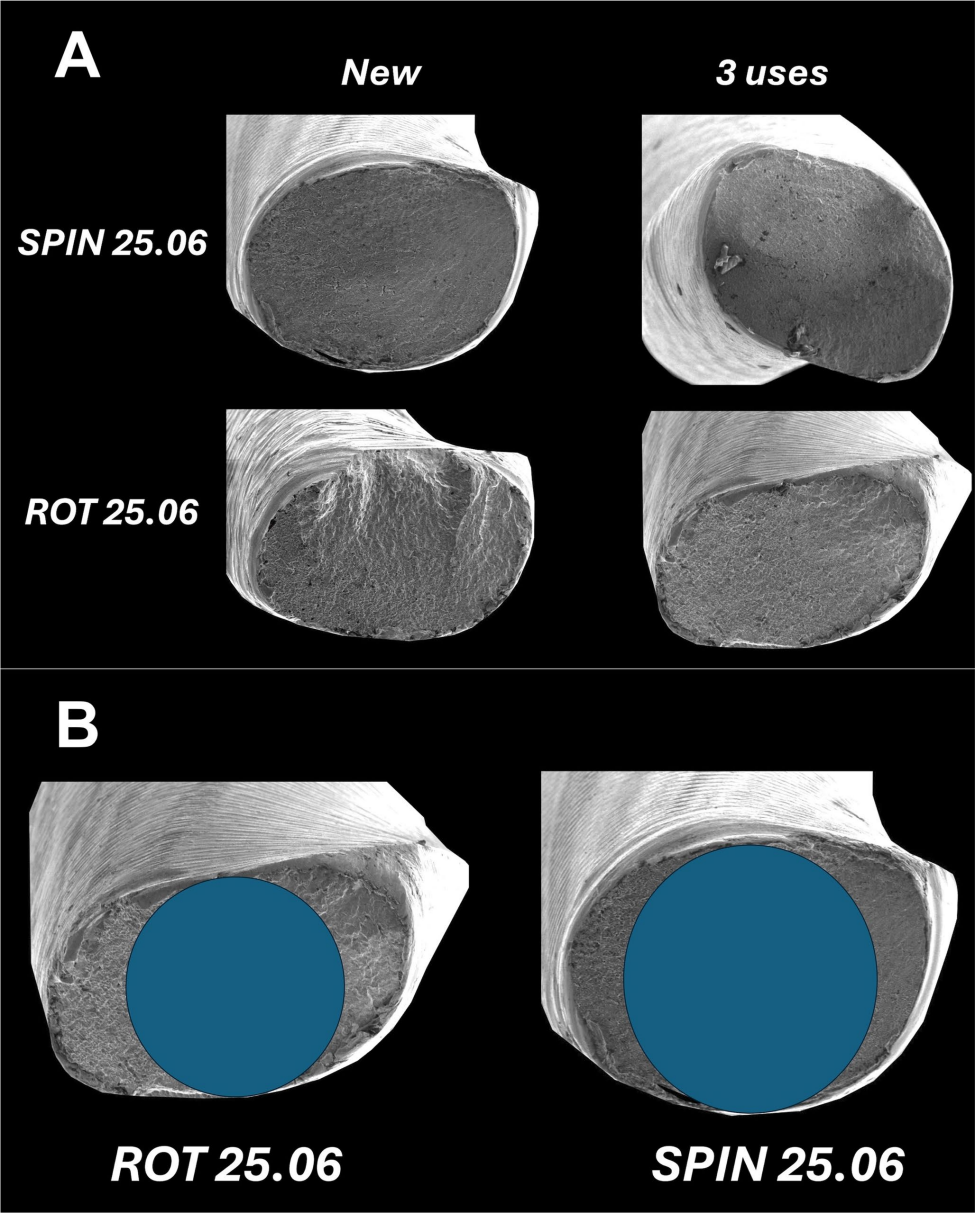
Scanning electron microscopy (SEM) analysis of the fractured surfaces (**A**) and representative core diameter and cross-sectional designs of the tested instruments (**B**)

The scanning electron microscopy (SEM) analysis of the fractured surfaces of all instruments revealed similar and typical features of cyclic fatigue failure. After the cyclic fatigue test, typical patterns were observed with micro voids, and characteristic morphological features of ductile fracture (Fig. 4A).

## Discussion

An important factor that should not be overlooked is the correct choice of NiTi instrument for each root canal anatomy, in terms of core diameter, taper, heat treatment of the alloy and file cross-section. It is a concern that a number of clinicians adopt a single system for all cases, as this can result in procedural errors [12, 24]. The challenge of treating root canals with complex or double curvatures causes the instruments to simultaneously undergo flexural and torsional stress and, therefore, requires instruments with a higher amount of R or martensite phase in their structure when working inside the root canal [8, 25] .

Both instruments used in this study exhibited similar blade design features (tip size, constant taper, length of active blade, number of spirals, cross-section design) and a smooth difference only in helical angle. However, the 2D (cross-sectional area) and 3D evaluation (metal mass volume) at 6 and 10 mm from the instrument tips demonstrated significant difference: Rotate 25.06 exhibited significantly lower metal mass volume and cross-sectional area than Spin 25.06 (*P* < 0.05). These results are extremely relevant, considering that the two instruments have an S-shaped cross-section, a constant taper, and the same tip size. Burklein et al. [23] demonstrated that core diameter can vary even among instruments with the same tip size, taper, and cross-section. The only explanation for this variation is related to manufacturing differences between the instruments. Therefore, despite their similar design features, the Spin instruments tend to exhibit greater metal mass volume and cross-section area along its cutting blades, which can impact on the mechanical properties and/or shaping ability [26, 27].

In this study, DSC analysis was performed to evaluate differences in the instruments’ phase transformation temperatures, as these temperatures directly affect phase transformation and thus influence instrument flexibility [12]. The results demonstrated that the As and Af temperatures were different between the instruments. Rotate 25.06 instruments would display a mixed microstructure of austenite and R-phase at room temperature (24 °C), whereas Spin 25.06 would predominantly present R-phase. On the other hand, at body temperature (36 °C), Rotate 25.06 instruments would display a fully austenitc microsctructure, while Spin 25.06 would present mixed microstructure of austenite and R-Phase. These findings were critical for demonstrating that the instruments exhibit different phase transformation behavior. In a study conducted to analyze the metallurgy and microstructure of instruments heat-treated or not, the former exhibited a prevalence of martensite grains that were stable at body temperature and consequently showed greater resistance to cyclic fatigue [26]. A recent critical review of the literature sobre NiTi instruments in endodontics states that these advantages, however, depend on instrument design, since taper, core mass, and cross-sectional geometry control the balance between cutting efficiency, torsional strength, and canal-centering ability [27].

The mechanical properties of NiTi instruments are essential for safe root canal preparation; however, these properties cannot be directly related to their shaping ability or overall safety during canal preparation [24, 28]. Therefore, multimethod studies have been widely employed to ensure a more comprehensive and effective analysis of the clinical application and performance of these instruments [13, 26].

The micro-CT evaluation demonstrated that Spin 25.06 promoted greater apical deviation and lower centering ability than Rotate 25.06. In addition, cyclic fatigue results indicated that new Spin instruments exhibited greater fatigue resistance than Rotate 25.06. However, after the preparation of three simulated double-curved canals, Spin instruments showed a significant reduction in fatigue resistance, whereas Rotate 25.06 maintained its fatigue performance. Previous studies demonstrated that design features (cross-section area, taper and core mass) and phase transformation temperature have strong effect on the mechanical properties and shaping ability of the NiTi instruments [12, 13, 24]. The higher phase transformation temperatures values of Spin 25.06 instruments should have favored a greater amount of R-phase or martensite and, consequently, improved canal center ability and reduced root canal deviation. On the other hand, these instruments exhibited a larger cross-sectional area e greater metal mass volume, which likely significantly reduced its flexibility and resulted in inferior shaping ability than Rotate 25.06.

In the cyclic fatigue test, Spin 25.06 demonstrated greater resistance to fatigue, suggesting it is more flexible than Rotate 25.06. This finding, however, differs from the results of the Micro-CT evaluation. The possible explanation can be attributed to the stainless-steel artificial canal presenting only a single curvature of the cyclic fatigue test, which generates a mechanical stress pattern distinct from that observed with double curvature. It’s likely that the use of double-curved artificial canal in the cyclic fatigue test, like that of the artificial block resin, could have resulted in different outcomes. This possibility can be observed in the cyclic fatigue results after the preparation of double-curved canals, as the Spin instruments exhibited a significant reduction in fatigue resistance, indicating that these instruments were subjected to greater mechanical stress during canal preparation. This likely occurred with the Spin 25.06 instrument due to its greater core diameter, which results in reduced flexibility and makes it more susceptible to fatigue after use [23]. It is also important to note that the cyclic fatigue resistance test was performed at room temperature, and that the results obtained would possibly be different at body temperature. In DSC calorimetry tests, Rotate instruments would display a mixed microsctructure of austenite and R-phase at room temperature and a fully austenitic microstructure at body temperature, and Spin instruments would predominantly present the R-phase at room temperature and mixed microstructure of austenite and R-Phase at body temperature. The austenite phase is characterized by stiffness, hardness, and increased superelasticity, and makes endodontic instruments less resistant to bending stress and cyclic fatigue [6].

A supplementary examination was conducted without statistical testing and used as a complementary evaluation to assess whether variations in metal mass volume and cross-sectional area were associated with differences in core diameter between the instruments. This analysis demonstrated that Rotate 25.06 presented a mean core diameter of 0.26 mm, whereas for Spin 25.06 this core diameter was 0.32 mm. Therefore, this supplementary examination confirmed our hypothesis regarding the greater core diameter of Spin instruments, which can be visually identified in a representative image (Fig. 4B).

The results of this study clearly demonstrate that the cyclic fatigue properties and metallurgical characteristics of NiTi mechanized instruments cannot always be directly associated with their shaping ability, as is commonly assumed. For this reason, studies evaluating only mechanical properties have not been published. Design-related factors, particularly core diameter, directly affect metal mass distribution and, consequently, modify both shaping performance and mechanical behavior. This relationship is clearly evidenced by the findings of the present study and is corroborated by other [12, 29]. Although the cross-section appears to greatly influence the cyclic fatigue resistance of instruments [29], this strong correlation is only observed when the characteristic is tested in isolation, yielding superior results for S-shaped cross-sections. A previous study [12] showed that, although instruments with the same cross-section, taper and tip size would typically have similar mechanical properties, they had significantly different resistance to cyclic fatigue, suggesting that other characteristics such as thermal treatment changes instruments’ crystallographic structure and strongly influenced cyclic fatigue resistance, while cross-sectional design primarily impacted torsional properties. Furthermore, instruments with lower flexibility tend to exhibit inferior shaping ability and experience greater mechanical stress during canal preparation, reducing their fatigue resistance and increasing the risk of fracture, as observed in the Spin group.

This study has significant limitations that should be considered before extrapolating its results to clinical practice. In this study, the shaping ability was evaluated using double-curved artificial canal in a resin block, as used in previous studies [30]. This variable was consistent across both groups, thereby ensuring standardized experimental conditions [31]. Although this allows standardization, it is important to emphasize that resin does not reproduce dentin hardness, instruments’ cutting behavior and clinical conditions. The absence of torsional fatigue testing presents another limitation. For future research, it is suggested that this property be compared among the instruments evaluated.

By integrating mechanical testing, analysis of metallurgical features, and instrument design evaluation, this approach offers a deeper insight into the differences between instruments of similar design. It combines both quantitative and qualitative techniques, thereby promoting triangulation and providing corroborative evidence. A key advantage is its ability to enhance research rigor, as traditional single-method studies, although valuable, often have limited scope and are prone to bias [13, 24].

## Conclusion

This in vitro study allows us to conclude that although Spin 25.06 demonstrated greater resistance to cyclic fatigue and higher phase transformation temperatures, Rotate 25.06 exhibited better shaping performance in artificial double-curved root canals, with reduced deviation and greater centering ability. These findings emphasize that heat treatment does not automatically outweigh the file parameters, particularly the core mass.

## Data Availability

No datasets were generated or analysed during the current study.
